# Annotating Putative *D. discoideum* Proteins Using I-TASSER

**DOI:** 10.17912/micropub.biology.000420

**Published:** 2021-07-14

**Authors:** Jacquelyn McCullough, Petra Fey, Ryan J. Rahman, Morgan Wallace, Seeta Morey, Kyle Sahlberg, Ethan McGonagle, Danielle Hess, Chance Hatfield, Michaela-Romina Sarmiento, Jordi Velasquez, Richard H. Gomer

**Affiliations:** 1 Department of Biology, Texas A&M University; 2 Center for Genetic Medicine, Northwestern University

## Abstract

Using Gene Ontology annotation in any aspect or using any evidence code, we found that approximately 14% percent of predicted *D. discoideum *proteins have no GO annotations and no obvious similarity to any annotated protein across diverse organisms. We have been systematically examining these unannotated protein sequences using software that predicts a protein structure and then compares the predicted structure to known structures.

**Figure 1. Example of a predicted structure used for annotating genes of unknown function:**
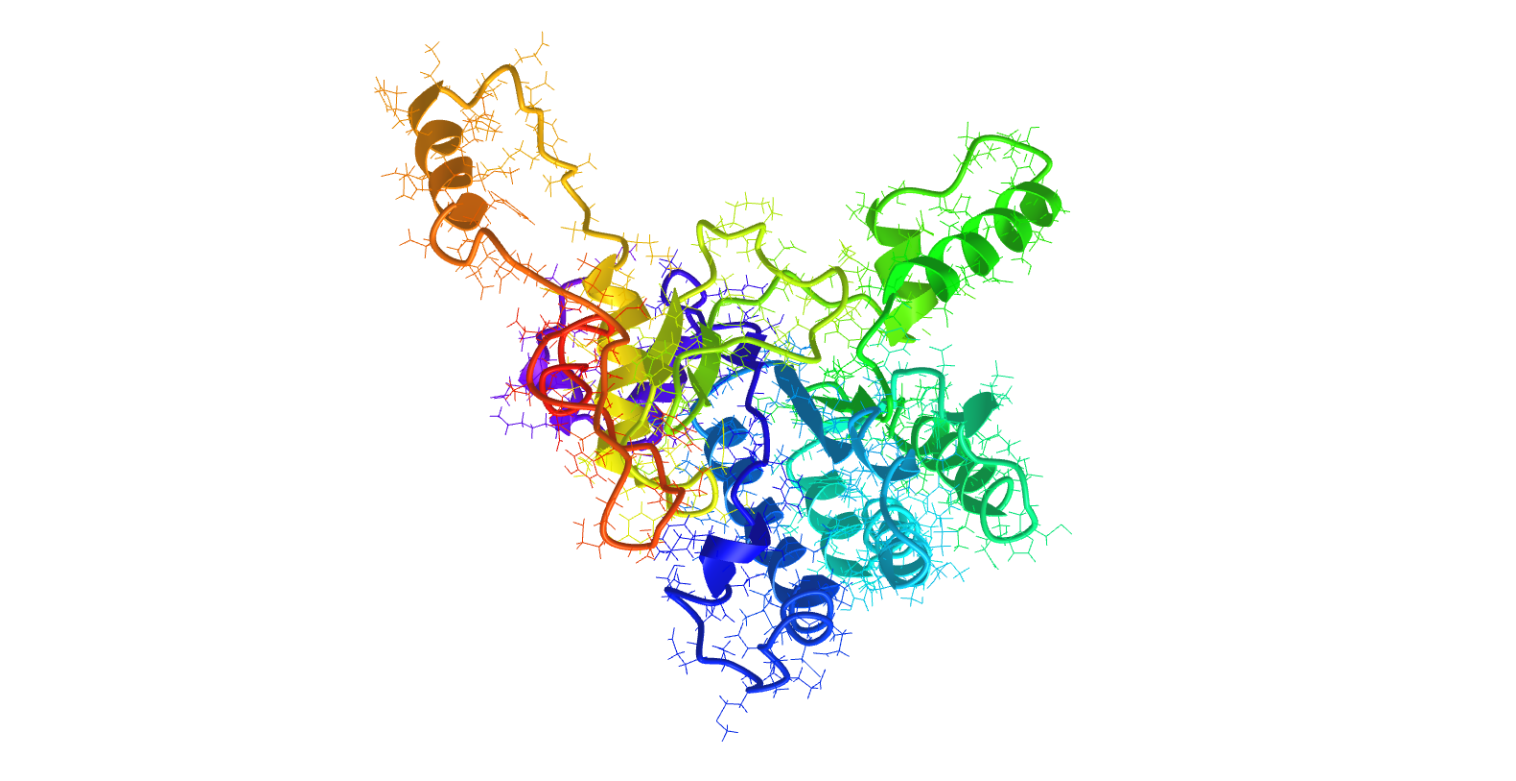
DDB_G0275279 annotations from I-TASSER. Image of top seeded protein model 1 with TM-score = 0.76±0.10. This model is structurally closest to PDB: 3wqm**,** a *Mycobacterium tuberculosis* hydrolase / hydrolase inhibitor, TM-score 0.888 and a coverage (Cov) of 0.901, which is the number of structurally aligned residues divided by the total protein length. The best Enzyme Commission hit is to PDB:2VG1, a *M. tuberculosis* transferase with TM-score 0.603, two mapped active residues, and EC number 2.5.1.68. Gene Ontology annotations include GO:0008834, di-trans,poly-cis-decaprenylcistransferase activity, GO-score 0.74; GO:0008360, regulation of cell shape, GO-score 0.74; GO:0051301, cell division, GO-score 0.74; GO:0009252, peptidoglycan biosynthetic process, GO-score 0.74; GO:0071555, cell wall organization, GO-score 0.74; and GO:0016094, polyprenol biosynthetic process, GO-score 0.55.

## Description

Although *D. discoideum* is an excellent model organism for many mammalian processes and disease states, large portions of its genome that could be useful for research remain uncharacterized. For instance, of the 11,088 protein coding genes in the *D. discoideum* genome that are not annotated as transposable elements or pseudogenes (Fey **et al.*,* 2019), the predicted amino acid sequence of 1,514 proteins are not similar enough to any known protein to receive a GO term, although many initiatives for experimental, large scale manual, and electronic annotations exist (Gene Ontology consortium, 2021).

An alternative way to predict the function of an amino acid sequence is to generate a predicted protein structure, and then compare the predicted structure to protein structures generated by X-ray crystallography or NMR. A commonly used program that does this sort of structure prediction and then structure comparison is I-TASSER (Roy **et al.*,* 2010; Yang **et al.*,* 2015; Zhang **et al.*,* 2017). I-TASSER generates secondary and tertiary structural predictions for a given protein, and then compares the predicted tertiary structure to known structures from the Protein Databank (PDB) (Burley **et al.*,* 2017). I-TASSER can then provide structure-based annotations for the protein, including gene ontology terms, possible ligand-binding, and enzymatic activity predictions. The 3D structures produced by I-TASSER can also be visualized and compared to Protein Databank proteins to, for instance, compare the active site of a protein in the PDB to a possible active site in the structure predicted by I-TASSER.

As an example of this approach, we previously found a *Dictyostelium* secreted protein called AprA that had very interesting properties, but unfortunately BLAST searches of the AprA amino acid sequence showed essentially no similarity to any mammalian proteins or possible gene products (comparing the AprA sequence to the amino acid sequences of all mammalian open reading frames) (Phillips and Gomer, 2012). I-TASSER predicted that AprA has similarity to the human secreted peptidase DPPIV, examination of the predicted AprA structure showed a very close match in both the amino acids and position of the amino acid side chains in one domain to a catalytic triad in the DPPIV structure, and DPPIV and AprA turned out to have very similar properties (Herlihy **et al.*,* 2013; Herlihy **et al.*,* 2017).

Over the course of a year, a group of 10 undergraduates was able to generate I-TASSER-based annotations for ~220 predicted *D. discoideum* proteins where there was no significant similarity to a known protein by GO or BLAST (although for many, there were similar hypothetical proteins in other organisms). Some of the I-TASSER identifications were to disparate proteins. For instance, the top structural analogs for the protein produced by *D. discoideum* gene DDB_G027895 included the BC component of the ABC complex from *Yersinia entomophaga* (TM score: 0.936), TedB2-TccC3 toxin from *Photorhabdus luminescens* (TM score: 0.872) and teneurin 2 partial extracellular domain (TM score 0.332). Another example of a protein with multiple possible similar proteins is encoded by DDB_G0275975; I-TASSER predicted that this protein has structural similarity to both antifreeze proteins and ABC transporters.

Other predicted proteins showed a single major similarity. For instance, the protein encoded by DDB_G0277585 has a predicted structural similarity to the ligand gated calcium channel inositol-1,4,5-triphosphate receptor (InsP_3_Rs), the proteins encoded by DDB_G0276051, DDB_G0277495, DDB_G0270742, and DDB_G0274535 all have predicted structural similarity to the *Clostridium difficile* toxins A and B, DDB_G027561 encodes a protein with predicted structural similarity to an influenza RNA-dependent RNA polymerase, DDB_G0276755 encodes a protein with predicted structural similarity to a toll like receptor 3 ligand binding domain, DDB_G0276921 encodes a protein with predicted structural similarity to a pilin proteins from the gram-positive bacteria Streptococcus Pyogenes, and DDB_G0276485 encodes a protein with predicted structural similarity to integrin cell adhesion proteins. In addition, some annotations revealed possible gene duplications. For instance, the proteins encoded by DDB_G0278559, DDB_G0278561, DDB_G0278565, and DDB_G0278553 all have predicted structural similarity to the MU2 Adaptin subunit in multiple species, and an examination of the chromosome location of these genes showed that they are all are near each other on chromosome 3.

As exemplified by I-TASSER correctly predicting a similarity between *D. discoideum* AprA and human DPPIV, we and others have found that structural similarities predicted by I-TASSER can be useful for annotations, especially in the case where sequence-based annotations do not return any useful information. In our initial set of annotations, we have found a wide variety of interesting and in some cases quite unexpected proteins, including what might be toxins and an RNA-dependent RNA polymerase. Additional I-TASSER studies on other proteins of unknown function, in both *D. discoideum* and other systems, may well yield additional surprises.

## Methods

We have begun to use I-TASSER to predict the possible function of the 1,514 predicted *Dictyostelium* proteins that have no functional annotations. To determine eligibility for I-TASSER analysis, we also did a BLAST search (Boratyn *et al.*, 2013) on the predicted amino acid sequence to ensure that it lacked significant sequence similarity to other known proteins. Once the BLAST search also returned no significant hits, the protein was then submitted to the online I-TASSER algorithm (https://zhanglab.dcmb.med.umich.edu/I-TASSER/). The I-TASSER analysis is time-intensive, and returns results in 4-7 days. Annotations were formatted primarily using the I-TASSER report and any information obtained from the preliminary BLAST search. Following the BLAST information, the top 3 PDB threading templates used by I-TASSER, top 5 predicted Protein Databank analogs, 3D structures, possible enzymatic activity, ligand binding, and GO terms were described in each annotation. A threading template is an amino acid sequence with a known fold that the LOMETS algorithms in I-TASSER use as a guide when generating 3D structures for the query protein. In contrast, the Protein Databank structural analogs are structures generated using X-ray crystallography and/or NMR which show similarity to the I-TASSER models of the query protein (I-TASSER often creates several possible protein structures).

The confidence measures in each annotation include the TM-score, the normalized Z-score, and the C-score. The TM-score measures topological similarity between a protein structure generated by I-TASSER and a pre-existing structure from Protein Databank. When comparing structures, the TM-score calculation weights smaller-distance mismatches less strongly than larger-distance mismatches for higher sensitivity. The TM-score has a range of 0 to 1, and a TM-score > 0.5 indicates a highly confident structural prediction. The normalized z-score is a measure of sequence alignment between the query protein and a threading template, which uses the number of conserved residues to determine whether the template is a good target. A normalized z-score > 1 indicates a good template. The C-score is a measure of predicted structure quality that ranges from -5 to 2, and a C-score > -1.5 typically indicates a correct fold. The C-score calculation accounts for both RMSD (Root Mean Square Deviation) of the predicted structure and normalized z-score of the template.

In addition to I-TASSER information, UniProt, PFAM, Interpro scan, and STRINGdb searches, as well as Kyte/Doolittle hydrophobicity plots, were used to enhance annotations. PFAM and Interpro provide information on protein superfamily membership and conserved functional domains for a protein query while STRINGdb compiles a web of linkages to the protein of interest such as predicted co-expression. Combining the information from these resources, each annotation aims to provide insight into a protein’s structure, function, and relevance to further research. I-TASSER utilizes the function database BioLiP (Yang, Roy *et al.,* 2013) to predict catalytic sites and has been extended to integrate functions from UniProtKB (UniProt consortium, 2021), Enzyme Commission (EC; (Bairoch, 2000)), and GO. The sequences of EC and GO were then mapped to BioLiP structural entries. In the Zhang group publications (Roy **et al.*,* 2010; Yang **et al.*,* 2015; Zhang **et al.*,* 2017), the authors provide metrics of several annotation aspects including GO, and they used a GO-score (0-1) cutoff at 0.5. This cutoff is a sensitive approach for GO annotations from I-TASSER.
